# IL-17A Expression Is Localised to Both Mononuclear and Polymorphonuclear Synovial Cell Infiltrates

**DOI:** 10.1371/journal.pone.0024048

**Published:** 2011-08-24

**Authors:** Ellen M. Moran, René Heydrich, Chin Teck Ng, Tajvur P. Saber, Jennifer McCormick, Joachim Sieper, Heiner Appel, Ursula Fearon, Douglas J. Veale

**Affiliations:** 1 Department of Rheumatology, St. Vincent's University Hospital, Dublin Academic Healthcare, Dublin, Ireland; 2 Charité Berlin, Berlin, Germany; Agency for Science, Technology and Research - Singapore Immunology Network, Singapore

## Abstract

**Introduction:**

This study examines the expression of IL-17A-secreting cells within the inflamed synovium and the relationship to *in vivo* joint hypoxia measurements.

**Methods:**

IL-17A expression was quantified in synovial tissue (ST), serum and synovial fluid (SF) by immunohistochemistry and MSD-plex assays. IL-6 SF and serum levels were measured by MSD-plex assays. Dual immunofluorescence for IL-17A was quantified in ST CD15+ cells (neutrophils), Tryptase+ (mast cells) and CD4+ (T cells). Synovial tissue oxygen (tpO_2_) levels were measured under direct visualisation at arthroscopy. Synovial infiltration was assessed using immunohistochemistry for cell specific markers. Peripheral blood mononuclear and polymorphonuclear cells were isolated and exposed to normoxic or 3% hypoxic conditions. IL-17A and IL-6 were quantified as above in culture supernatants.

**Results:**

IL-17A expression was localised to mononuclear and polymorphonuclear (PMN) cells in inflamed ST. Dual immunoflourescent staining co-localised IL-17A expression with CD15+ neutrophils Tryptase+ mast cells and CD4+T cells. % IL-17A positivity was highest on CD15+ neutrophils, followed by mast cells and then CD4+T-cells. The number of IL-17A-secreting PMN cells significantly correlated with sublining CD68 expression (r = 0.618, p<0.01). IL-17A SF levels correlated with IL-6 SF levels (r = 0.675, p<0.01). Patients categorized according to tp0_2_< or >20mmHg, showed those with low tp0_2_<20mmHg had significantly higher IL-17A+ mononuclear cells with no difference observed for PMNs. Exposure of mononuclear and polymorphonuclear cells to 3% hypoxia, significantly induced IL-6 in mononuclear cells, but had no effect on IL-17A expression in mononuclear and polymorphonuclear cells.

**Conclusion:**

This study demonstrates IL-17A expression is localised to several immune cell subtypes within the inflamed synovial tissue, further supporting the concept that IL-17A is a key mediator in inflammatory arthritis. The association of hypoxia with Il-17A expression appears to be indirect, probably through hypoxia-induced pro-inflammatory pathways and leukocyte influx within the joint microenvironment.

## Introduction

Rheumatoid arthritis (RA) and Psoriatic arthritis (PsA) are the most common forms of the inflammatory rheumatic diseases characterised by synovitis and progressive destruction of articular cartilage and bone [1], [2]. Angiogenesis is a primary crucial step in disease pathogenesis which facilitates the recruitment and migration of inflammatory cell types into the inflamed joint cavity [3]. Subsequently, the synovial lining layer thickens and the sublining is infiltrated with T cells, B cells, mast cells, neutrophils, monocytes and macrophages which secrete a wide range of mediators which further exacerbate the inflammatory response [4], [5], however little is known about the role of mast cells in driving the inflammatory response.

Mast cells have been implicated in IgE-mediated immune responses in the context of allergic disease and defence against helminths [6], [7]. Recent studies in the K/BxN mouse model however, have firmly established mast cells as having a critical role in the pathogenesis of inflammatory arthritis [8], [9]. These findings have renewed interest in previous histological studies demonstrating a marked increase in mast cell expression in the human RA synovial sublining, in particular at sites of cartilage erosion, and their relationship to increased joint inflammation [7], [10]. Furthermore, mast cell derived mediators such as tryptase have been implicated in the activation of synovial fibroblasts and proteoglycan depletion [11].

Targeted biologic agents in particular TNF inhibitors (TNFi) have advanced the treatment of both RA and PsA, although some patients do not respond highlighting the need for alternative therapies. The pro-inflammatory cytokine IL-17A is one such potential target. IL-17A is the first identified member of the IL-17 family (A–F), it is most closely related to IL-17F with 50% sequence homology [12]. IL-17A has been localized to T-cell rich areas in the RA synovium and overexpression of IL-17A has been detected in serum and SF samples from inflammatory arthritis patients compared to OA and healthy control subjects [13], [14], [15], [16]. Furthermore, expression of IL-17A correlates with disease activity and clinical response that can be modulated by both DMARD and TNFi therapy [13], [17]. In humans Th17 cells are a key source of IL-17A when activated by a number of key cytokines required for their development including TGF-β, IL-6, IL-21, IL-1 and IL-23 [18]. In addition to Th17 cells, γδT, NK, NKT and innate immune cells such as mast cells and neutrophils have been identified as sources of IL-17A in murine studies [19] and more recently in humans [20]. IL-17A, alone and in combination with other proinflammatory cytokines, drives ECM remodelling and cartilage destruction through the induction of MMPs [13].

Hypoxia has been implicated in RA pathogenesis, previous studies have demonstrated that the level of oxygen in SF from patients with RA is reduced compared to healthy controls. Low oxygen levels have been reported in tenosynovium from RA patients with tendon rupture [21]. More recently we have demonstrated profound hypoxia in inflamed ST using an oxygen sensing probe *in vivo,* levels of which were inversely associated with synovial inflammation and blood vessel morphology [22], [23] Several studies using synovial cells, have shown that hypoxia may induce key angiogenic growth factors (VEGF and Angiopoietins), chemokines (MCP-1, IL-8, MIP-3α) and MMPs −1, −3 and −9 while downregulating IL-10 [23], [24]. Furthermore sustained hypoxia activates NF-κB dependent gene expression, which is a key regulator of inflammation genes [25]. Together this data highlights the ability of hypoxia to regulate diverse signalling pathways that are involved in the pro-inflammatory response.

In this study we demonstrate that IL-17A is expressed by important immune cells including mast cells within the inflamed synovium. Furthermore, we demonstrate a relationship between *in vivo* measures of hypoxia and IL-17A producing cells in the inflamed joint; however it is unclear whether this effect is direct or indirect.

## Materials and Methods

### Patient recruitment

Patients with active inflammatory arthritis, RA and PsA, were recruited from outpatient clinics at Department of Rheumatology, St. Vincent's University Hospital (SVUH). All patients fulfilled diagnostic criteria for RA or PsA [26], [27]. This study was approved by St. Vincent's University Hospital institutional ethics committee, and all patients gave their fully informed written consent prior to inclusion in the study. All patients had active disease despite being on disease modifying anti-rheumatic drugs (DMARDs), had at least one inflamed knee joint and were commencing on biologic therapy. Clinical and laboratory assessment included tender and swollen joint count, rheumatoid factor, anti-CCP antibody (ACPA), erythrocyte sedimentation rate (ESR), C-reactive protein (CRP) and global health visual analogue scale (VAS) obtained on the same day as the arthroscopy.

### Arthroscopy, oxygen measurements and sample collection

Arthroscopy of the inflamed knee was performed under local anaesthetic using a Wolf 2.7 mm needle arthroscope, biopsies were obtained from inflamed synovial membrane and oxygen partial pressure was measured under direct visualization as previously described [23]. For future immunohistochemical analysis synovial biopsies were embedded in OCT (Tissue Tek, The Netherlands) and stored at −80°C or paraffin embedded. Serum (n = 28) and matched synovial fluid (n = 19) were collected immediately before arthroscopy and stored at −80°C.

### IL-17A Immunohistochemistry

IL-17A immunohistochemistry was performed using 3 µm paraffin ST sections. The sections were deparaffinised in xylene and rehydrated in alcohol and deionised water. Antigen retrieval was performed by heating sections in solution (10mM citrate buffer, pH 6.0) in a pressure cooker. Slides were washed with TBS-Triton 3 times and one time with TBS for 5 minutes. Non-specific binding was blocked using Dako Protein Block (Dako, Glostrup, Denmark) for 5 minutes. The blocking buffer was then removed and sections were incubated with a polyclonal anti-IL-17A antibody (R&D Systems, Wiesbaden-Nordenstadt, Germany). Control experiments were performed with (i) an isotype control; and (ii) an anti-IL-17A antibody block using recombinant IL-17A (R&D Systems). After incubation overnight at 4°C and washing, the sections were incubated with biotinylated rabbit anti-goat IgG antibody diluted in Dako Real Antibody Diluent (Dako) for 30 minutes at room temperature, followed by strepavidin-peroxidase complex (Dako, Glostrup, Denmark) for 30 minutes at room temperature. Following washing the slides were visualised by reacting for 10–15 minutes with Fast Red Substrate-Chromagen System (Dako). Nuclear counterstaining was performed using Mayer's haematoxylin; the sections were then dehydrated, and mounted in glycerol gelatine. For the quantification of IL-17A expressing cells in the RA and PsA synovium, 10 high power fields (HPFs) of one section were analysed per patient and the absolute number was divided by 10 to obtain the average number of those cells per HPF. Cell specific markers for CD68 and CD3 cells were previously quantified in this cohort [23]. In brief sections were incubated with primary antibodies against mouse-monoclonal anti-CD68, anti-CD3, (DAKO, Glostrup, Denmark) at room temperature for 1 hour. A routine three-stage immunoperoxidase labelling technique incorporating avidin-biotin-immunoperoxidase complex (DAKO, Glostrup, Denmark) was used. Colour was developed in solution containing diaminobenzadine-tetrahydrochloride, counterstained with haematoxylin and mounted. Slides were analysed using a well established semi-quantitative scoring method ranging from 0–4 (0 =  no staining, 1 = <25%, 2 = 25–50%, 3 = 50–75%, 4 = >75% staining)[23] .

### IL-17A Co-localisation to Synovial cells

Immunohistochemistry of paraffin embedded ST was performed to detect tryptase+ mast cells with a monoclonal anti-human antibody (clone AA-1) and CD15+neutrophils with an anti-CD15 antibody (clone MMA, Acris, Herfordt, Germany). Dual immunofluorescent staining was performed to identify IL-17A expressing cells using a polyclonal anti-IL-17A antibody. Sections were prepared as above then incubated overnight with primary antibodies to (1) goat polyclonal anti-IL-17/rabbit polyclonal Myeloperoxidase (Thermo/Lab Vision), (2) goat polyclonal anti-IL-17/mouse monoclonal anti-Mast cell-Tryptase (DAKO, UK) and (3) goat polyclonal anti-IL-17/mouse monoclonal CD4. Sections were then washed and incubated with secondary antibodies against, Donkey anti-rabbit AlexaFluor 488 (invitrogen) or donkey anti-mouse Alexa Fluor 488 (Invitrogen) and Donkey anti-goat Nothern Light 577 (RnDsystems, UK). Slides were counterstained with Dapi nuclear stain and mounted with antifade fluorescent mounting media. The scoring was initially performed by microscope analysis at high power and then dual immunoflourescent staining was performed for validation. IL-17A, CD4, CD15 and MPO quantification was assessed in ten random high-powered fields (HPF) of one section per patient. Specificity of the IL-17A antibody was further tested by pre-incubation of this antibody with recombinant human IL-17A in blocking experiments, as well as by obtaining the expected pattern of IL-17, CD4, CD15 and MPO-staining in the positive control tissues, tonsil and lymph node. All the immunofluor- doublestains were performed in the positive control tissues. Furthermore isotype-matched control antibodies for IL-17, CD4, CD15 and MPO, were performed as negative controls.

### Cytokine measurement by Multiplex Assay

The MSD multiplex assay was used to quantify IL-17A in synovial fluids and culture supernatants. IL-6 in synovial fluids and culture supernatants was quantified by MSD multiplex or ELISA (R&D systems, Cambridge, UK). The samples and standards were prepared on multiplex assay plates or ELISA plates in accordance with the manufacturer's instructions. The Sector Imager 2400 instrument and software were used to read the multiplex plate, and absorbance was measured at 450nm in a microtiter plate spectrophotometer (Dynatech MR4000, Alexandria, VA) for ELISA.

#### Isolation of peripheral blood mononuclear cells (PBMCs) and Neutrophils

Peripheral blood mononuclear cells (PBMCs) from healthy donors were isolated by Ficoll-Metrizoate density gradient centrifugation (Lymphoprep; Nycomed, UK). Human neutrophils were purified from healthy donors by dextran sedimentation and Ficoll gradient centrifugation followed by hypotonic lysis of contaminating erthryocytes. Neutrophils were resuspended in 1% EGM medium prior to experiments. Cells were seeded in 98-well plates, at a cell density of approximately 500,000 cells/ml in full RPMI 1640 medium, and cultured under 3% hypoxic or normoxic conditions for 24hrs. Supernatants were harvested and IL-17 and IL-6 were quantified by MSD assays and ELISA.

### Statistical Analysis

SPSS12 system for windows was used for statistical analysis. Non-parametric Wilcoxon Signed Rank test, Mann-Whitney U test and Spearman correlation coefficient were used for analysis of non-parametric data. p<0.05 was determined as statistically significant.

## Results

### Localized production of IL-17A within the inflammatory joint

**Figure 1:**
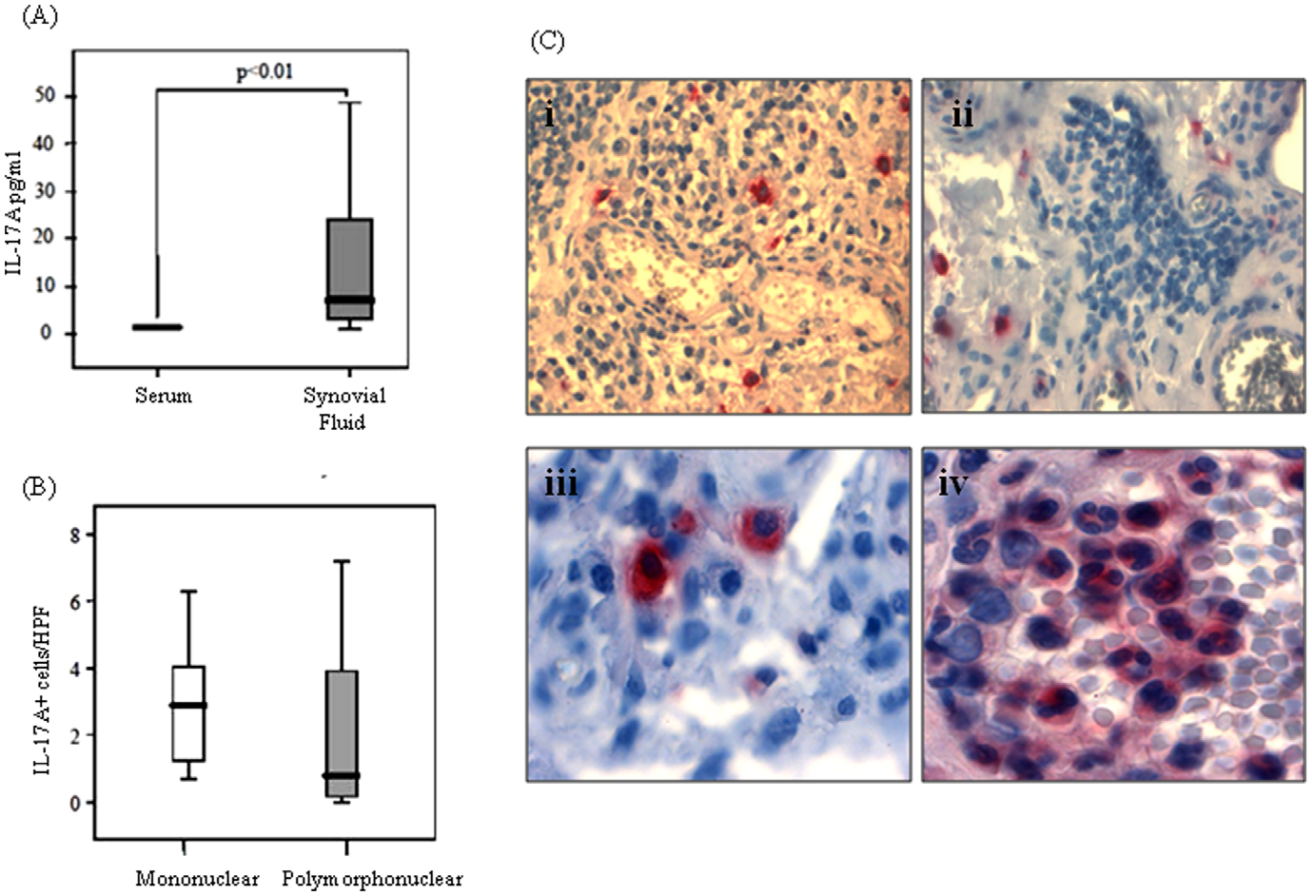
IL-17A expression is localized to the inflamed joint. IL-17A protein levels were measured by MSD Assay in paired serum/synovial fluid samples (n = 20) (**A**). Synovial fluid levels were significantly higher than serum levels. Values expressed as median ± range, *p<0.01, significance level. Immunohistochemistry was performed in synovial tissue sections from patients with inflammatory arthritis (n = 19). (**B**).The number of mononuclear cells (white bars) staining for IL-17A was higher than the number of IL-17A positive polymorphonuclear cells (grey bars). Results are expressed as the number of IL-17A positive cells per high powered field (HPF). (**C**) Representative images of IL-17A expression in RA (i) vs. PsA (ii) and mononuclear IL-17A expression (iii) and polymorphonuclear IL-17A expression (iv).

Serum and SF samples (n = 22) were analyzed by IL-17A MSD assay. Serum IL-17 levels were [1.5pg/ml (0.8–2.8)]. When we compared matched serum and SF levels, we demonstrated that SF IL-17A levels [6.9pg/ml (0.9–156)] were significantly higher than serum IL-17A levels [1.5pg/ml (0.8–2.8)] (p<0.001) (Figure 1A). Immunohistochemical analysis of IL-17A in ST sections detects IL-17A positive cells in the sublining layer of all patients; no lining layer expression was observed (Figure 1B and C). IL-17A immunostaining was observed in both mononuclear and polymorphonuclear (PMN) cells (Figure 1C). The number of IL-17A positive cells in each patient were quantified (Figure 1B). The number of IL-17A positive mononuclear cells tended to be higher compared to PMN cells [2.9 cells/HPF (0.7–11.4) vs. 0.8cells/HPF (0–18.10)]. IL-17A positive cells were then categorized by patient diagnosis – RA or PsA .RA patients (n = 11) had more IL-17A positive mononuclear than PMN cells [2.7cells/HPF (0.7–11.4) vs. 1.3 cells/HPF (0–7.2)]. A similar trend was also observed for PsA patients (n = 8) [2.9 cells/HPF (0.7–6.30) vs. 0.7 cells/HPF (0–18.1)]. Furthermore no significant differences were observed in IL-17A positive cell numbers between RA and PsA cohorts. Representative images of IL-17A positive cells in RA (i) vs. PsA (ii) and high power images of IL-17A on MNCs (iii) and PMN (iv) are shown in Figure 1C.

### Production of IL-17A by the innate immune cells – mast cells and neutrophils

**Figure 2:**
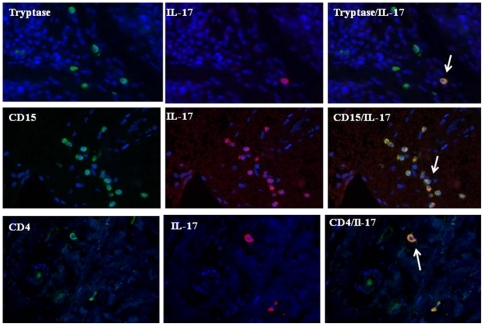
Localisation of IL-17A to neutrophils and mast cells within the inflamed synovium. Representative images of RA synovial tissue section stained with antibodies against tryptase, CD15 and IL-17A. Merged images indicating co-localisation – yellow.

Dual-immunofluorescence staining was performed to identify the precise phenotype of the IL-17A positive PMN cells identified within the inflamed synovium. Using dual immunofluorescence staining we demonstrate IL-17A positive cells co-localised with tryptase identifying IL-17A positive mast cells (Figure 2A) and CD15 neutrophils (Figure 2B). Consistent with previous work we also show IL-17A positive cells co-localised with CD4+ T cells (Figure 2C). Table 1 shows the % IL-17A positivity on the different cell types. Percentage was quantified by (i) % double staining positivity of whole IL-17A cell count or (ii) % double staining of tryptase +, CD15+ or CD4+ cells. Table 1 shows that using both quantifications, IL-17A+ expression was highest on neutrophils, mast cells and CD4 T cells respectively.

**Table 1:** Results are expressed as mean (standard deviation) percentage.

	Percentage of cell subtypes expressing IL-17+/ total IL-17+ cells	Percentage of IL-17+ expressing cells/ cell subtype
Tryptase+/analysis (n = 10)	27.36 (29.09)	78.91 (12)
CD15+/analysis (n = 10)	81.08 (29.8)	78.91(29.9)
CD4+/analysis (n = 10)	1.28 (4.86)	1.65 (2.54)

### Intra-articular pO2 levels and IL-17A production

**Figure 3:**
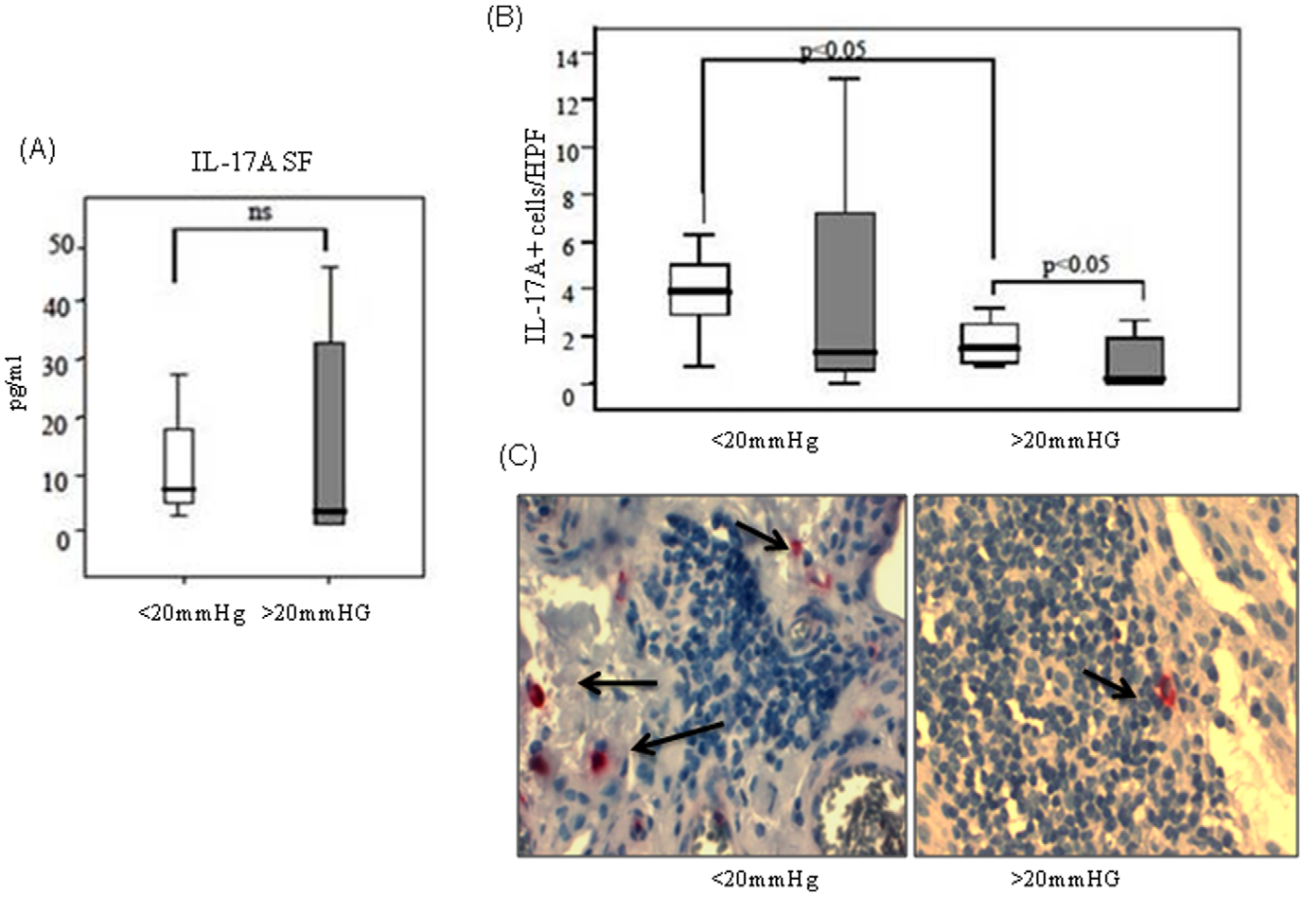
Increased systemic expression of IL-17A at low pO2. (A) Patient synovial fluid samples (n = 22) were assessed by MSD assay for the expression of IL-17A. Cytokine levels were then grouped according to patient tpO_2_ levels or >20mmHg. No significant difference in synovial fluid levels was observed between the two groups. (B) Synovial tissue pO_2_ levels (n = 18) were also examined in relation to the expression of IL-17A positive mononuclear (white bars) and polymorphonuclear cells (grey bars). Patients with tpO_2_ levels <20mmHg (n = 9) had significantly more IL-17A positive mononuclear cells than those with tpO_2_ levels >20mmHg (n = 9) (p<0.05). Patients with tpO_2_ levels <20mmHg (n = 9) also had a higher number of IL-17A positive polymorphonuclear cells than those with tpO_2_ levels >20mmHg (n = 9). This difference was not statistically different. (C) Representative images of IL-17A expression on mononuclear cells from a patient with high tpO2 levels vs a patient with low tpO2 levels are shown.

Synovial tissue pO_2_ was quantified using a Licox probe and demonstrated profound levels of hypoxia within the joint as recently described [22], [23], [28], [29]. When patients were grouped into those with low pO_2_ (<20mmHg) or those with high pO_2_ (>20mmHg) as previously described (23), higher levels of IL-17A positive mononuclear and PMN cells were associated with lower tpO2, no difference was observed in SF levels of IL-17A (Figure 3A,B). Patients with tpO_2_ <20mmHg had significantly more IL-17A positive mononuclear cells than those with tpO_2_>20mmHg [3.9 cells/HPF (0.7–6.3) vs. 1.7 cells/HPF (0.7–11.4)] (p<0.05) (Figure 3B). Patients with tpO_2_ <20mmHg also had a higher number of IL-17A positive PMN cells than those with tpO_2_ levels >20mmHg [1.3 cells/HPF (0–18.1) vs. 0.15 cells/HPF (0–5.10), however this did not reach significance. IL-17A positive mononuclear cells were significant higher than IL-17A positive PMN cells in patients with tpO_2_ levels >20mmHg (p<0.05) (Figure 3C). When PMNs were examined separately for CD15+IL-17A+ and Tryptase+IL-17A+ cells, higher levels of CD15+IL-17A+ were demonstrated in patients with tpO2 <20mmHg, however no significant difference was observed for mast cells.

### IL-17A and IL-6 expression and inflammatory infiltrate

The relationship of IL-17A expression within the inflammatory joint with markers of inflammation and cellular infiltrate was assessed. Levels of IL-17A correlated with SF IL-6 levels (r = 0.675, p<0.01). SF IL-17A levels also correlate with the CD3+ T cell infiltration in the ST lining layer (r = 0.545, p<0.05). The relationship between IL-17A positive cells and inflammatory cellular infiltrate was also assessed. Neither cell type correlated with CD3+ T cell expression however the expression of IL-17A PMN cells correlated with the sublining expression of CD68+ macrophages (r = 0.618, p<0.01).

### The effect of hypoxia on IL-17A production in PBMC and neutrophil cultures

**Figure 4:**
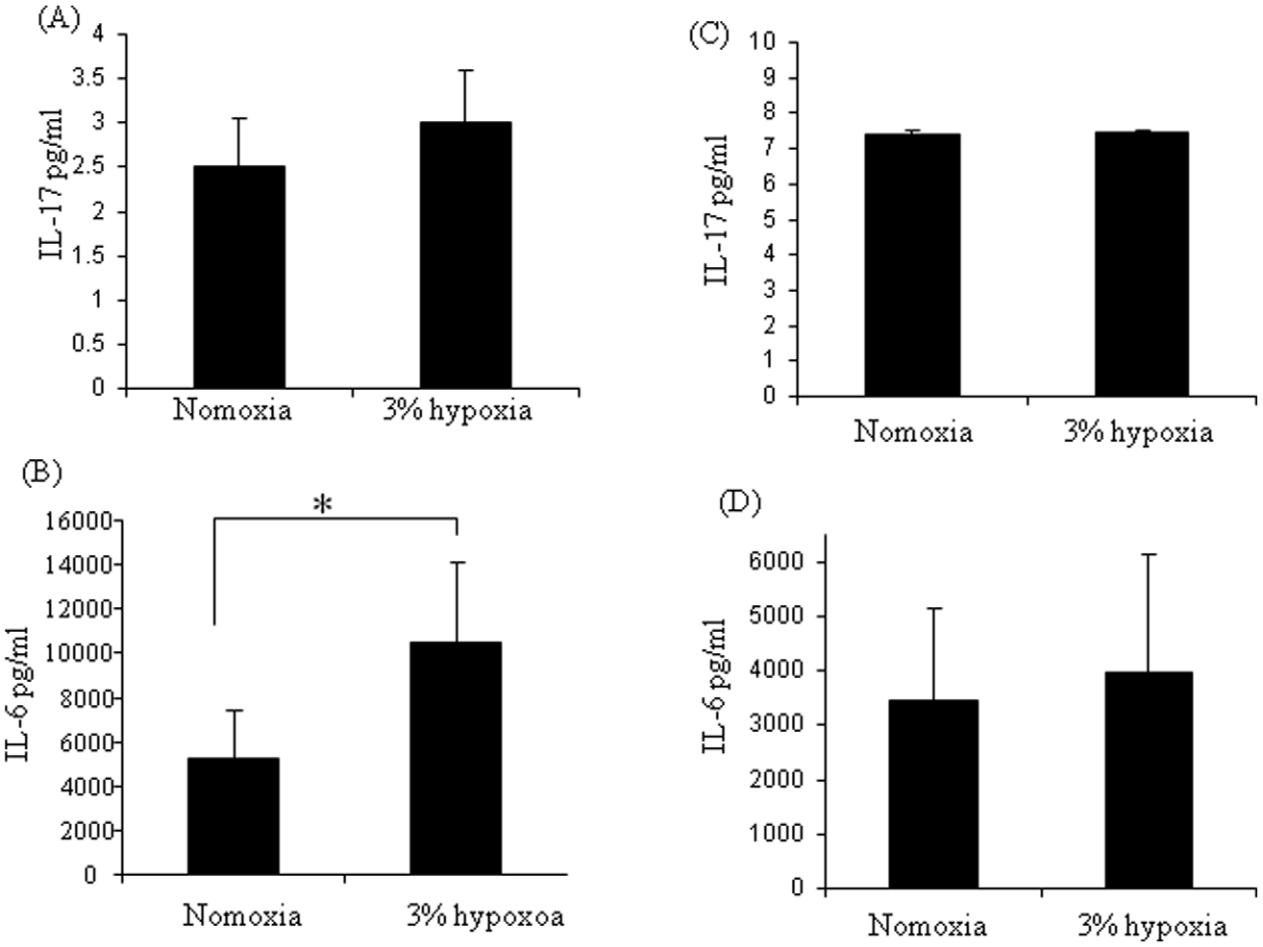
The effect of 3% hypoxia on IL-17A and IL-6 *in vitro*. Peripheral blood mononuclear cells (n = 12) and neutrophils (n = 6) were cultured for 24hrs under 3% hypoxic conditions and normoxia. Hypoxia had no effect on IL-17A expression (A), but significantly induced IL-6 expression in mononuclear cells (B). Hypoxia had no effect on IL-17A(C) or IL-6 (D) in neutrophils. Data expressed as the mean ± SEM. P<0.05 significantly different from normoxic conditions.

IL-17A was measured in mononuclear cell and neutrophils following exposure to 3% hypoxia. No significant difference in IL-17A levels under normoxic or hypoxic conditions was observed (Figure 4 A, C). In contrast IL-6 levels were significantly induced in mononuclear cells (Figure 4B) (p<0.05) with no significant difference observed in neutrophils (Figure 4D).

## Discussion

In this study we demonstrate *in vivo* the presence of IL-17A expressing -neutrophils, mast cells and T–cells within the inflamed synovium. Percentage positivity of IL-17A was highest on neutrophils, followed by mast cells and then CD4+T cells. We demonstrate that IL-17A is highly expressed in the inflamed joint and is associated with the expression of IL-6 and inflammatory cell infiltrate. Furthermore, we demonstrate tissue mononuclear cell expression of IL-17A is significantly higher in patients with low *in vivo* tissue pO_2_ levels. Finally no difference in IL-17A levels was observed following exposure to hypoxia *in vitro*. Expression of IL-17A on CD15+neutrophils and tryptase+ mast cells in addition to CD4+T-cells further supports the concept that IL-17A plays a key role in the pathogenesis of inflammatory arthritis. This association with hypoxia, is most likely an indirect effect due to induced infiltration of inflammatory immune cells into the synovial pannus [22], [28].

IL-17A expression is significantly higher in inflammatory arthritis SF compared to serum levels, suggesting IL-17A production is predominantly localized within the joint consistent with our previous findings [13]. Furthermore, IL-17A expression within the joint has been shown to strongly correlate with disease activity and inflammation [13], [30]. Immunohistochemical analysis of ST from inflammatory arthritis patients demonstrated sublining expression of IL-17A, particularly in areas of lymphoid infiltration. In previous reports these cells were mainly mononuclear [16] although we now demonstrate, IL-17A+ synovial PMN cells co-localizing IL-17A with tryptase+ mast cells and CD15+ neutrophils. Murine mast cells and neutrophils have been previously shown to express IL-17A following specific stimulation; however, it has not been well established in human tissue [30], [31]. Furthermore, these cells have are known to be a key source of proinflammatory cytokines in human RA ST [31], and interact with RA synovial fibroblast cells *via* the production of soluble mediators to enhance IL-6 secretion [32]. Here we demonstrate mast cells and neutrophils expressing IL-17A within the inflamed synovium. Both cell types have been implicated in the pathogenesis of CIA and other models of experimental arthritis [33], [34], [35].

Our data supports Hueber et al [20], who demonstrated the majority of IL-17A expressing cells in RA synovial tissue were co-localised to mast cell [20]. Furthermore they showed that pro-inflammatory stimuli such as TNFα,C5 and LPS alone and in combination induce RORC-dependant IL-17A production from mast cells *in vitro*. This data suggests that mast cells are a major source of IL-17A in the inflamed synovium, which can be induced by the pro-inflammatory microenvironment of the joint. In our study we showed that IL-17A expression was localized to tryptase+ mast cells, CD15+ neutrophils and CD4+T cells, with highest expression observed on CD15+ neutrophils. The expression of IL-17A on several cell subtypes within the synovium, suggest it plays an important immune-modulatory role. Our data is consistent with recent reports in psoriasis skin biopsies showing similar IL-17A expression patterns [36], [37].

Furthermore, IL-17A^+^ PMN cells correlated with sublining CD68 expression which again supports previous studies that demonstrate mast cells can activate resident synovial macrophages *via* the production of various proinflammatory mediators and recruit both neutrophils and monocytes into the joint. Mast cells and neutrophils have both been implicated in the initiation and progression of arthritis [10], [38]. IL-17A is a well established mediator of angiogenesis and inflammatory cell influx *via* the production of cytokines and chemokines [39], [40], [41]. The expression of IL-17A by these cells further implicates the pivotal role IL-17A plays in the pathogenesis of RA.

In addition we demonstrated that *in vivo* measures of hypoxia were associated with synovial mononuclear IL-17A expression. This is supported by previous studies demonstrating the effect of hypoxia on immune cells. Studies in both human and murine tissue have shown T cell accumulation in hypoxic tissue [42], [43], and its expression has been previously shown to be associated with hypoxia. Exposure of murine CD3+ T cells to hypoxia enhances T cell expression, proliferation and activation in a HIF-1α dependent manner. We have previously shown that low hypoxia is inversely associated with synovial mononuclear cell infiltrates [23], vascularity [22] and others have shown that HIF1α is co-localised to synovial mononuclear cells in the joint [44], and with potent chemotactic factors macrophage inflammatory protein CCL20 (MIP3α) and Stromal cell derived factor-1 (SDF-1) and angiogenesis [45]. Hypoxia enhances amyloid beta peptide induced IL-17A production and T_H_-17 differentiation in PBMC cultures [46]. Normally neutrophils have a short half-life and rapidly undergo apoptosis; however following exposure to hypoxia neutrophil apoptosis can be suppressed [47], [48], [49].

While we found no increase in the number of IL-17A+ mast cells in patients with low tpO2 levels, previous studies suggest mast cells respond early to hypoxic insult in rat models of cerebral ischemia [50], [51]. Exposure to hypoxia has increased production of MMPs and tryptase by mast cells leading to tissue degradation [52]. The expression of IL-17A and its receptor are upregulated in both murine and human ischemic tissue compared to non ischemic tissue [53]. Murine mast cells have been shown to produce IL-17A in response to stimulation with TLR2 ligands [54]. Furthermore, human mast cells have been shown to stimulate activated T cells suggesting a potential role in T_H_-17 differentiation. Mast cells have been shown to be early responders to a hypoxic insult and degranulation of mast cells can be detected histologically 1–2 hours after the initiation of arthritis in the K/BxN model [8], [50], [51], [52].

In this study while IL-17A expression was associated with low pO2 levels and hypoxia induced IL-6 expression *in vitro*, no effect on IL-17A expression *in vitro* was observed. This suggests that the association between hypoxia and IL-17 is indirect and possibly due to the effect of hypoxia on several pro-inflammatory pathways and influx of inflammatory immune cells into inflamed joint. Hypoxia did induce IL-6 levels in monocyte, suggesting that hypoxia induces differential cytokine signaling pathways which may depend on cell-type. This is consistent with our previous work in which we demonstrated that patients with low *in vivo* measures of tpO_2_ were significantly associated with high CD3+T cells and CD68 macrophages infiltrates and increased expression of TNFα, IL-1β, IFN-γ and MIP-3α [23]. The effect of hypoxia on pro-inflammatory mediators has been demonstrated by several *in vitro* studies [55], [56], [57] showing induction of TNFα, IL-1β, VEGF. Induction of macrophage inflammatory protein CCL20 (MIP-3α) in SF monocytes and ICAM in lymphocytes following exposure to hypoxia has also been demonstrated [23], [50], [58]. Whether hypoxia is driving the increase of IL-17A expression in the joint or whether it is due to increased inflammation is unclear. The association of hypoxia with inflammatory cells and MIP-3α induction would support a role for a hypoxia-induced influx of inflammatory immune cells as MIP-3α is involved in attracting IL-17A positive cells to the joint. However, several studies have suggested that T_H_-17 cells do not acquire a fully activated phenotype until they are resident within the inflammatory joint [59], [60] where the presence of soluble mediators and cell-cell interactions influence their differentiation [18], [61], [62], [63].

In conclusion we have localised IL-17A expression to neutrophils and mast cells in inflamed human synovium, with highest positivity demonstrated on neutrophils. The expression of IL-17A in the serum, SF and tissue of inflammatory arthritis patients was associated with inflammation and cellular infiltrate. While no direct relationship between hypoxia and IL-17A production was established, hypoxia may influence IL-17A expression by upregulating production of various soluble mediators [23], in addition to induction of leukocyte influx into the synovium inflammatory processes.
